# Effects of sustained weight loss on outcomes associated with obesity comorbidities and healthcare resource utilization

**DOI:** 10.1371/journal.pone.0258545

**Published:** 2021-11-03

**Authors:** G. Craig Wood, Lisa Bailey-Davis, Peter Benotti, Adam Cook, James Dove, Jacob Mowery, Abhilasha Ramasamy, Neeraj Iyer, B. Gabriel Smolarz, Neela Kumar, Christopher D. Still

**Affiliations:** 1 Geisinger Health, Danville, Pennsylvania, United States of America; 2 Novo Nordisk Inc, Plainsboro, New Jersey, United States of America; McMaster University, CANADA

## Abstract

**Objective:**

Determine the impact of long-term non-surgical weight loss maintenance on clinical relevance for osteoarthritis, cancer, opioid use, and depression/anxiety and healthcare resource utilization.

**Methods:**

A cohort of adults receiving primary care within Geisinger Health System between 2001–2017 was retrospectively studied. Patients with ≥3 weight measurements in the two-year index period and obesity at baseline (BMI ≥30 kg/m^2^) were categorized: Obesity Maintainers (reference group) maintained weight within +/-3%; Weight Loss Rebounders lost ≥5% body weight in year one, regaining ≥20% of weight loss in year two; Weight Loss Maintainers lost ≥5% body weight in year one, maintaining ≥80% of weight loss. Association with development of osteoarthritis, cancer, opioid use, and depression/anxiety, was assessed; healthcare resource utilization was quantified. Magnitude of weight loss among maintainers was evaluated for impact on health outcomes.

**Results:**

In total, 63,567 patients were analyzed including 67% Obesity Maintainers, 19% Weight Loss Rebounders, and 14% Weight Loss Maintainers; median follow-up was 9.7 years. Time until osteoarthritis onset was delayed for Weight Loss Maintainers compared to Obesity Maintainers (Logrank test p <0.0001). Female Weight Loss Maintainers had a 19% and 24% lower risk of developing any cancer (p = 0.0022) or obesity-related cancer (p = 0.0021), respectively. No significant trends were observed for opioid use. Weight loss Rebounders and Maintainers had increased risk (14% and 25%) of future treatment for anxiety/depression (both <0.0001). Weight loss maintenance of >15% weight loss was associated with the greatest decrease in incident osteoarthritis. Healthcare resource utilization was significantly higher for Weight Loss Rebounders and Maintainers compared to Obesity Maintainers. Increased weight loss among Weight Loss Maintainers trended with lower overall healthcare resource utilization, except for hospitalizations.

**Conclusions:**

In people with obesity, sustained weight loss was associated with greater clinical benefits than regained short-term weight loss and obesity maintenance. Higher weight loss magnitudes were associated with delayed onset of osteoarthritis and led to decreased healthcare utilization.

## Introduction

Obesity is a chronic disease that has been associated with a multitude of comorbidities, including cardiovascular disease, diabetes, certain cancers, joint diseases, mental health disorders, and sleeping disorders [1–11] with a negative impact on quality of life [12]. As the prevalence of obesity continues to rise to more than 40% of the U.S. adult population [13], the health and societal effects will be substantial.

Weight loss achieved through a variety of lifestyle interventions, anti-obesity medications, or bariatric surgery can improve health outcomes and reduce the risk of mortality [14–20]. However, weight loss achieved through caloric restriction or bariatric surgery has many limitations including effects on basal metabolic rate and endocrine regulation, as well as frequent reoperations needed with bariatric surgery [21–26] which can make it challenging for many people with obesity to sustain weight loss over time [27, 28].

There is little in the literature describing the longer-term clinical impact of weight loss with regain and sustained weight loss on obesity [29]. The aim of this study was to determine the relationship between long-term weight loss maintenance and clinical relevance across a range of comorbidities that can particularly impact the patients with obesity including osteoarthritis, cancer, opioid use, and depression/anxiety. In another report, we have summarized outcomes related to the cardiometabolic comorbidities of type 2 diabetes, hypertension, and cardiovascular disease (article in preparation). Time to development of each condition and the effect of varying magnitudes of weight loss was assessed in a broad patient population which sought care at a large integrated healthcare delivery system in the United States (U.S.) over a ten-year period. A secondary objective was to examine the relationship between obesity and long-term weight loss maintenance on health care resource utilization.

## Methods

### Study population

A retrospective observational study was performed with patients receiving primary care at Geisinger Health System between 2001 and 2017. The Geisinger Institutional Review Board reviewed the study, determined it qualified for exempt status, and granted a waiver of patient consent. Geisinger Health System is a Pennsylvania-based integrated delivery system (IDS) which includes a health plan, acute care hospitals, specialty hospitals, ambulatory surgery centers, and additional clinical services [30]. There are numerous research units within the Geisinger Health System including the Obesity Institute, which provides resources supporting obesity research across the IDS [31].

The study population included adult patients who were at least 18 years of age and for whom three or more weight measurements were documented in the electronic health record (EHR) over a two-year period. This is denoted as the index period and included a baseline weight, a one-year weight (within 6–18 months), and a two-year weight (within 12–24 months). The index period was preceded by a lead-in period to establish medical history. Weight measurements within 15 months prior to baseline BMI measurement were excluded. Outcomes were observed following the index period only and weight changes were not analyzed following the index period. Any patient who had bariatric surgery prior to or during the index period or prevalent/history of cancer were excluded from the study. The weight measurements within six-months of pregnancy indicators were also excluded for women who were pregnant during the index period. Based on weight trends during each year of the index period, three study groups with a history of obesity were defined: 1) *Obesity Maintainers*: patients who maintained weight within ±3% margin from baseline; 2) *Weight Loss Rebounders*: patients who lost ≥5% weight via non-surgical methods and regained weight 20% or more of one-year weight loss from baseline (weight regain of 20% or more was selected as a boundary based on the King et al. study exploring weight regain measurements [32]); and 3) *Weight Loss Maintainers*: patients who lost ≥5% weight via non-surgical methods and maintained ≥80% of the one-year weight loss from baseline. All patients were censored at the time of the last visit in the health record.

To determine if health outcomes were impacted by the magnitude of weight loss, the Weight Loss Maintainers group was stratified by amount of initial weight loss (i.e., <7%, 7–10%, >10–15%, and >15%). Only patients meeting the definition of any of the three groups were included in the analysis.

### Study outcomes

Outcomes were analyzed post-index period and included a range of physical and mental domains of health. The outcomes reported in this study included osteoarthritis, cancer, opioid use, and depression/anxiety. Cardiometabolic outcomes have been reported elsewhere (article in preparation). Outcomes associated with osteoarthritis were defined by EHR documentation of International Classification of Diseases 10^th^ edition (ICD-10) diagnosis codes on the problem list or at least two outpatient visits. Time until osteoarthritis was calculated as a new occurrence of an osteoarthritis diagnosis. Osteoarthritis diagnosis was defined as ICD-10 (ICD-10 M15-M19) on problem list or 2+ outpatient visits. Cancer diagnosis was defined as any in situ or malignant condition as previously defined by the Pennsylvania Cancer Registry and Commission on Cancer, categorized by ICD-10 code for the primary cancer site. To be included in the Geisinger tumor registry, cases are either diagnosed and/or treated for the condition within the Geisinger Health System. Analyses were conducted for all documented cancer types and then for a subset of cancer types that have been associated with obesity: breast, uterine, colon, kidney, pancreas, thyroid, liver, rectum, stomach, esophagus, ovary, gallbladder, and rectosigmoid junction (S1 Table) [8–10]. Time until cancer was calculated as a new occurrence of cancer in the Geisinger Health System tumor registry. Since males and females are predisposed to different cancer types, the analyses for time until cancer were stratified by sex. Opioid use was included as a proxy for pain. Time until opioid use was defined as a new occurrence of opioid use and was defined as two or more outpatient prescriptions for opioids. Time until treatment for depression/anxiety was defined as medication orders or active use of a depression/anxiety medication occurring after the end of the index period; medications included alpha-2 receptor antagonists (tetracyclics), benzodiazepines, monoamine oxidase inhibitors, selective serotonin reuptake inhibitors, serotonin modulators, serotonin-norepinephrine reuptake inhibitors, and tricyclic agents.

The classification of prevalence and incidence of each condition was evaluated based on the timing of the first signal that occurred within the index period; specifically, patients who met the diagnostic or treatment criteria prior to or during the index period were considered as having prevalent comorbidity, whereas those who met diagnostic criteria after the index period were considered as having incident comorbidity. To capture only incident disease, patients with prevalent disease were excluded from analysis related to that disease. For example, patients with prevalent osteoarthritis were excluded from the analysis that identified emergence of osteoarthritis. Data on weight measurements, socio-demographics, vital signs, laboratory tests, encounters, procedures, diagnostic codes, orders (pharmacological, diet, etc.) was extracted from Geisinger’s EPIC^®^ EHR and data warehouse. Median height was calculated and used for all body mass index (BMI) measures. Weight measurements of <80 pounds and >700 pounds and height measurements of <42 inches and >90 inches were excluded as outliers.

**Fig 1:**
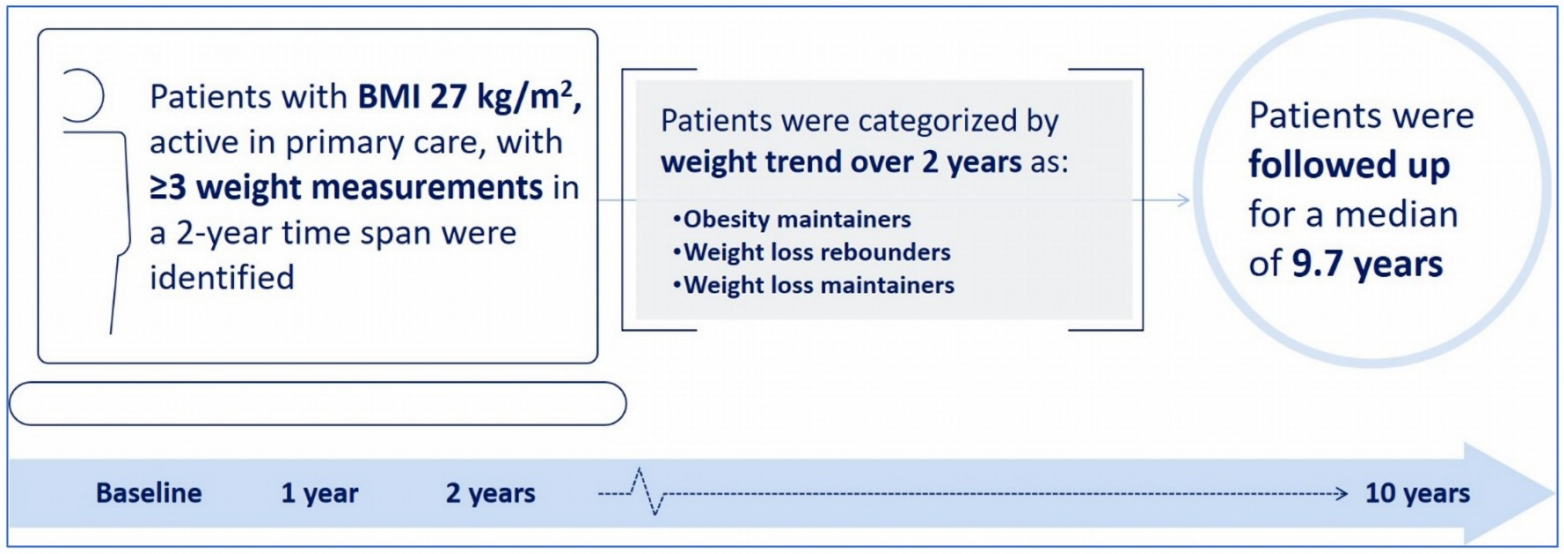
Overview of study design and timeline. BMI, body mass index.

A lead-in period to establish medical history was used by excluding weight measurements during the first 15 months that patients participated in primary care within the Geisinger Health System (Fig 1). To allow for adequate time to capture three EHR-recorded weight measures, specific ranges were defined. Time zero, or the beginning of the index period was defined as a baseline weight of BMI ≥30 kg/m^2^. A second weight measurement occurred approximately one year after baseline assessment within 6–18 months, followed by a third weight measurement at least one year later, within 12–24 months. This approximates a baseline, year one, and year two weight measurement. A follow-up visit was conducted at least 6 months after the last weight measure.

Overall healthcare utilization was characterized using three encounter types: 1) outpatient visits, 2) emergency department (ED) visits, and 3) hospitalizations. The encounters were defined and calculated as follows: total number of days with an outpatient encounter (limited to those involving contact with a care provider); total number of days with an ED visit and time until first ED visit; and total number of hospitalizations, number of hospitalized days, and time until first hospitalization. Utilization was limited to visits occurring within the Geisinger Health System’s clinics and hospitals.

#### Statistical analyses

Independent and joint associations of weight loss and weight maintenance on each clinical outcome were evaluated. To ensure that the final complex models were representative of associations found within underlying analysis, the statistical analyses proceeded from simple to complex, starting with descriptive statistics and unadjusted analysis, followed by adjusted regression modeling.

The simple analyses evaluated the unadjusted association of each clinical indictor with the weight loss groups (Obesity Maintainers, Weight Loss Rebounders, and Weight Loss Maintainers) using Cox regression for dichotomous outcomes (i.e., a time-to-event regression model). For each clinical indicator, time-to-outcome was calculated as the number of days between the initial baseline weight measurement until the outcome of interest occurred. For patients who did not develop the outcome of interest, the time was censored at the last follow-up visit. Testing for proportional hazard assumptions allowed for the examination of consistent effects in the short-term and the long-term.

The unadjusted analyses were followed by models that adjusted for selected patient characteristics and tested whether these characteristics influenced the effect of weight loss on the clinical outcomes using Obesity Maintainers as the reference group. The final models presented in this paper were adjusted for age, sex, BMI, diabetes, hypertension treatment, hyperlipidemia treatment, depression/anxiety treatment, osteoarthritis, asthma, and gastrointestinal reflux disease (GERD). The Charlson Index, a validated score combining multiple comorbidities into a 10-year survival predictor [33] was also used as a covariate as were other conditions not included in the Charlson Index. Comorbidities were based on diagnosis codes documented in the EHR and weighted higher for diseases with greater mortality risk based on the Charlson Index.

The cumulative incidence of osteoarthritis, cancer, time to opioid use, depression/anxiety were estimated by the Kaplan-Meier (KM) method and plotted over 10 years of follow-up for each patient sub-group. Additionally, Kaplan-Meier curves were used to compare time until outcome with the Weight Loss Maintainers group stratified by the amount of weight loss at the end of year 2 of the index period: <7%, 7–10%, >10–15%, and >15%. A minimum weight loss of 7% was examined based on research demonstrating the effect of weight loss of at least 7% preventing or delaying development of type 2 diabetes [34]. Sensitivity analyses were conducted to determine if the results were influenced by the amount of weight loss during the first year or the amount of weight regain from year one to year two. Patients diagnosed with the outcome of interest prior to baseline or during the index period were excluded from the analysis. The analyses were conducted using SAS version 9.4 software.

Healthcare resource utilization was compared between groups using Poisson Regression. These analyses were conducted using unadjusted raw data and after adjusting for age, sex, BMI, Charlson Index, and selected/prevalent baseline comorbidities (diabetes, hypertension treatment, hyperlipidemia treatment, osteoarthritis, depression/anxiety treatment, asthma, and GERD).

## Results

### Study population

The final study sample comprised of 63,567 patients, classified as Obesity Maintainers (67%), Weight Loss Rebounders (19%), and Weight Loss Maintainers (14%) with a median follow-up period of 9.7 years. Baseline descriptive statistics and disease status for the study population are presented in Table 1.

**Table 1:** Baseline descriptive statistics of the study population.

	Obesity Maintainers (n = 42,534)	Weight Loss Rebounders (n = 12,227)	Weight Loss Maintainers (n = 8,806)	p-value
	Mean (SD)			
**Age (years)**	53.3 (14.5)	47.9 (15.4)	50.1 (17.2)	<0.0001
**BMI (kg/m^2^)**	35.3 (5.4)	35.9 (6.1)	35.8 (6.2)	<0.0001
	n (%)			
**Sex, % female**	22,136 (52.0)	7,600 (62.2)	5,838 (66.3)	<0.0001
**Race/ethnicity**	n (%)			
White	41,337 (97.2)	11,855 (97.0)	8,510 (96.7)	0.096
Hispanic	544 (1.3)	171 (1.4)	141 (1.6)	
Black	522 (1.2)	159 (1.3)	132 (1.5)	
Asian	59 (<0.1)	21 (<0.1)	9 (<0.1)	
Hawaiian	26 (<0.1)	7 (<0.1)	1 (<0.1)	
American Indian	24 (<0.1)	9 (<0.1)	5 (<0.1)	
Other/unknown	22 (<0.1)	5 (<0.1)	8 (<0.1)	
**Disease status**	n (%)			
Type 2 diabetes	7,175 (16.9)	1,709 (14.0)	1,658 (18.8)	<0.0001
Pre-diabetes	5,981 (14.1)	1,445 (11.8)	1,059 (12.0)	<0.0001
Treatment for hyperlipidemia	13,853 (32.6)	3,025 (24.7)	2,527 (28.7)	<0.0001
Treatment for hypertension	20,166 (47.4)	4,775 (39.1)	3,899 (44.3)	<0.0001
Any cardiovascular disease	10,007 (23.5)	2,516 (20.6)	2,252 (25.6)	<0.0001
Congestive heart failure	903 (2.1)	258 (2.1)	307 (3.5)	<0.0001
Stroke	57 (0.1)	20 (0.2)	22 (0.3)	0.042
Myocardial infarction	483 (1.1)	121 (1.0)	93 (1.1)	0.364
Osteoarthritis	8,107 (19.1)	2,141 (17.5)	1,813 (20.6)	<0.0001
Treatment for depression/anxiety	12,489 (29.4)	4,488 (36.7)	3,437 (39.0)	<0.0001
Sleep apnea	2,539 (6.0)	782 (6.4)	549 (6.2)	0.183
Asthma	4,118 (9.7)	1,484 (12.1)	1,089 (12.4)	<0.0001
Gastroesophageal reflux disease	10,147 (23.9)	2,942 (24.1)	2,254 (25.6)	0.0023
Charlson Index = 0	28,753 (67.6)	8,442 (69.0)	5,488 (62.3)	<0.0001
Charlson Index = 1	10,177 (23.9)	2,757 (22.6)	2,229 (25.3)	
Charlson Index = 2+	3,604 (8.5)	1,028 (8.4)	1,089 (12.4)	

SD, standard deviation; BMI, body mass index

Specific weight loss interventions including visits with a weight loss specialist, visits with a registered dietitian or nutritionist, or anti-obesity medication use were documented for only a small portion of patients. Patients who received/utilized any of these weight loss treatments included 2.5% of Obesity Maintainers, 4.5% of Weight Loss Rebounders, and 3.9% of Weight Loss Maintainers.

### Impact of obesity on health-related outcomes

**Fig 2:**
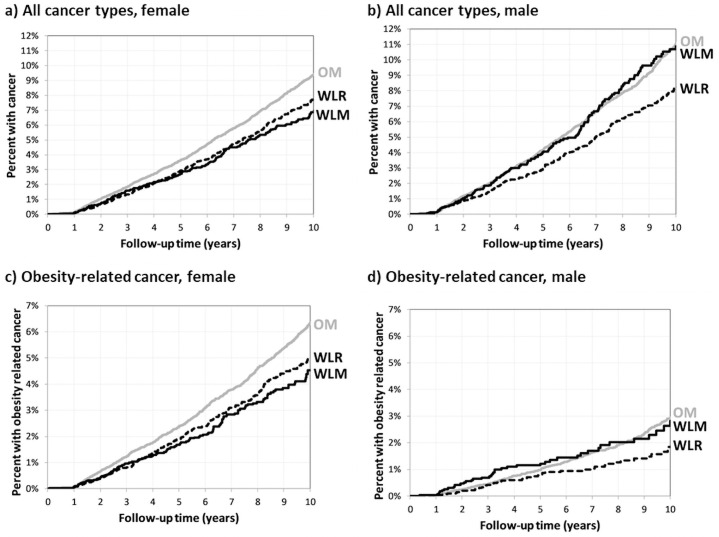
Kaplan-Meier curves for time until cancer stratified by sex and OM, WLR, and WLM. A. All cancer types, female. B. All cancer types, male. C. Obesity-related cancer, female. D. Obesity-related cancer, male. OM, Obesity Maintainers; WLR, Weight Loss Rebounders; WLM, Weight Loss Maintainers.

The adjusted hazard ratios for the risk associated with developing the studied outcomes are displayed in Table 2. Weight Loss Maintainers had the longest time until an osteoarthritis diagnosis and Obesity Maintainers had the shortest time (Logrank test p <0.0001). Compared to Obesity Maintainers, Weight Loss Maintainers had a lower risk of incident osteoarthritis (Hazard Ratio, HR = 0.904); the difference between Obesity Maintainers and Weight Loss Rebounders was not significant. Females in the Weight Loss Maintainers group had a 19% lower risk of developing any cancer (p = 0.0022) and a 24% lower risk of developing obesity-related cancer (p = 0.0021) compared to those in the Obesity Maintainers group (Fig 2 and S1 Appendix). There were no significant differences in time to developing any type of cancer or obesity-related cancer among males.

**Table 2:** Adjusted Cox regression model for time until health-related outcomes.

Outcome^a^	Weight trend group sample size^b^	Comparison	Adjusted HR	95% CI	p-value
Osteoarthritis	OM: 31,397 WLR: 9,380 WLM: 6,493	WLR vs. OM	0.974	[0.913, 1.040]	0.432
		WLM vs. OM	0.904	[0.837, 0.977]	0.011
Cancer	OM: 42,534 WLR: 12,227 WLM: 8,806	Females: WLR vs. OM	0.994	[0.887, 1.115]	0.923
		WLM vs. OM	0.808	[0.704, 0926]	0.0022
		Males: WLR vs. OM	0.914	[0.795, 1.049]	0.201
		WLM vs. OM	1.026	[0.876, 1.203]	0.748
Obesity-Related Cancer		Females: WLR vs. OM	0.969	[0.842, 1.116]	0.666
		WLM vs. OM	0.764	[0.644, 0.907]	0.0021
		Males: WLR vs. OM	0.835	[0.630, 1.106]	0.209
		WLM vs. OM	1.069	[0.785, 1.456]	0.672
Opioid use	OM: 25,835 WLM: 4,425 WLR: 6,567	WLR vs. OM	1.061	[1.020, 1.103]	0.003
		WLM vs. OM	1.030	[0.983, 1.078]	0.213
Depression/Anxiety	OM: 25,068 WLR: 5,763 WLM: 4,031	WLR vs. OM	1.143	[1.092, 1.197]	<0.0001
		WLM vs. OM	1.247	[1.183, 1.314]	<0.0001

^a^ Models were adjusted for age, sex, BMI, Charlson Index, diabetes, hypertension treatment, hyperlipidemia treatment, depression/anxiety treatment, osteoarthritis, asthma, and GERD.

^b^ Excluding those with incident outcomes prior to baseline or during the index period; pre-existing cancer was an exclusion criterion, no patients with pre-existing cancer were excluded from this analysis.

OM, Obesity Maintainers; WLR: Weight Loss Rebounders; WLM, Weight Loss Maintainers.

**Fig 3:**
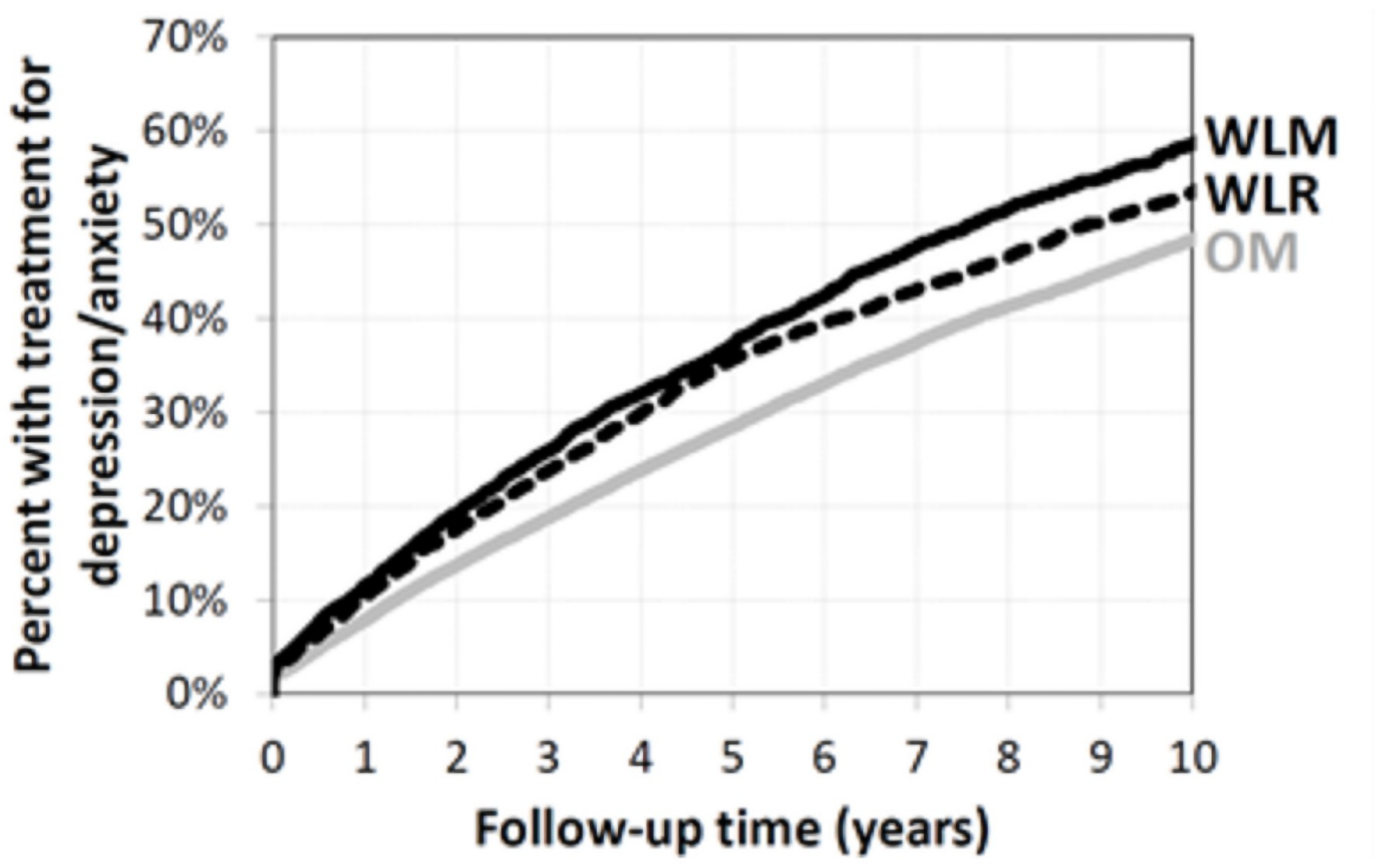
Kaplan-Meier curves for time until depression/anxiety stratified by OM, WLR, and WLM. OM, Obesity Maintainers; WLR, Weight Loss Rebounders; WLM, Weight Loss Maintainers.

Compared to Obesity Maintainers, Weight Loss Rebounders and Weight Loss Maintainers had a 14% and 25% higher risk of future treatment for depression/anxiety, respectively. Weight Loss Maintainers had the shortest time until treatment for depression/anxiety and Obesity Maintainers had the longest time until treatment for depression/anxiety (Logrank test p<0.0001) (Fig 3 and S1 Appendix).

### Impact of magnitudes of weight loss on health-related outcomes

**Fig 4:**
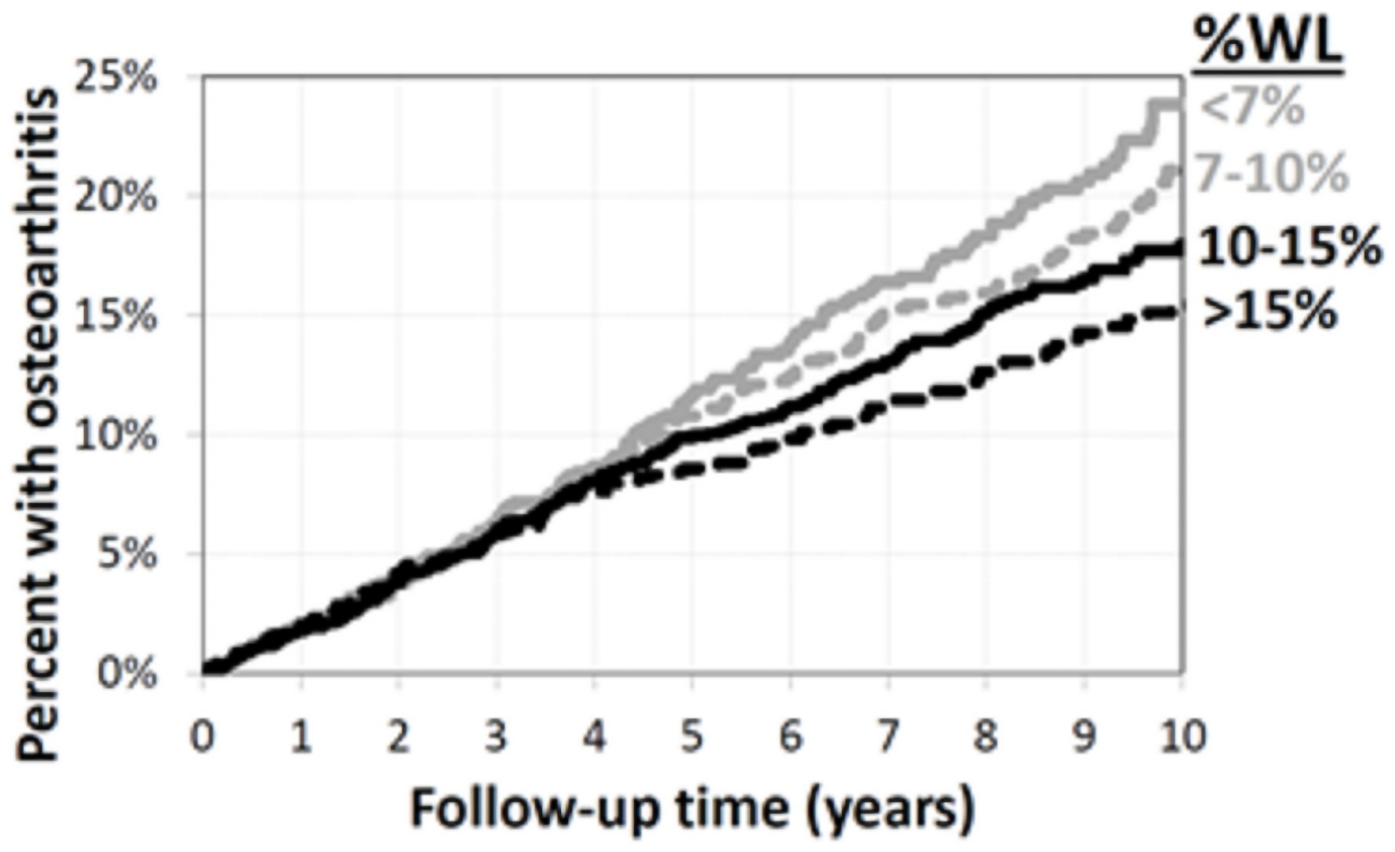
Kaplan-Meier curves for time until osteoarthritis within the WLM group stratified by the amount of weight loss at the end of the 2+ year index period. WLM, Weight Loss Maintainers; WL, weight loss.

There was a significant effect modification of time associated with the relationship between amount of weight loss and time until osteoarthritis (failed the proportional hazards assumption). The Kaplan-Meier curve suggests a delayed effect signified by little difference between groups early in follow-up and diverging curves later in follow-up (Fig 4 and S1 Appendix). Thus, the adjusted Cox models were adapted to account for the time-varying covariate of weight loss amount. Specifically, the adjusted hazard ratios were calculated for the first four years of follow-up (where there was little difference between the weight loss groups) and then again for four years of follow-up onward (where the differences between weight loss groups were apparent). Compared to those who had <7% weight loss, patients with >15% weight loss had a 47% lower risk of incident osteoarthritis (p = 0.0006) starting at four years of follow-up.

There was no association with the amount of weight loss in the Weight Loss Maintainers group and time until depression/anxiety or cancer in either sex. Although the association trended in the direction of decreased opioid use with increasing weight loss, there was no significant association.

### Healthcare resource utilization

Healthcare resource utilization was significantly higher for Weight Loss Maintainers and Weight Loss Rebounders compared to the Obesity Maintainers for outpatient visits, ED visits, hospitalizations, and inpatient days (Table 3). Additionally, Weight Loss Maintainers and Weight Loss Rebounders had a significantly shorter time to ED visit as compared to Obesity Maintainers: HR = 1.106 for Weight Loss Maintainers, (p = 0.0001) and HR = 1.062 for Weight Loss Rebounders (p = 0.0094). Similar trends were seen for hospitalizations: HR = 1.158 for Weight Loss Maintainers (p <0.0001) and HR = 1.095 for Weight Loss Rebounders (p = 0.0003).

**Table 3:** Poisson regression for days with healthcare utilization visits per year compared between study groups.

Healthcare utilization type	Obesity Maintainers	Weight Loss Rebounders	Weight Loss Maintainers
	n = 42,534	n = 12,227	n = 8,806
Outpatient visits			
Mean total outpatient visits (SD)	38.0 (35.5)	36.9 (35.0)	36.5 (35.5)
Mean follow-up years (SD)	6.74 (3.92)	6.47 (3.83)	6.13 (3.83)
Outpatient visits per year of follow-up	5.64	5.70	5.96
Adjusted relative risk	Ref	1.036	1.045
		[1.032, 1.039]	[1.041, 1.049]
[95% CI], p-value^a^		<0.0001	<0.0001
Emergency department (ED) visits			
Mean total ED visits (SD)	0.40 (1.41)	0.50 (2.04)	0.53 (1.88)
Mean follow-up years (SD)	6.74 (3.92)	6.47 (3.83)	6.13 (3.83)
ED visits per year of follow-up	0.059	0.078	0.086
Adjusted relative risk	Ref	1.156	1.237
		[1.122, 1.191]	[1.197, 1.279]
[95% CI], p-value^a^		<0.0001	<0.0001
Inpatient (hospital) visits			
Mean total inpatient visits (SD)	0.35 (1.04)	0.35 (1.21)	0.39 (1.25)
Mean number inpatient days (SD)	1.34 (5.21)	1.40 (6.06)	1.59 (6.54)
Mean follow-up years (SD)	6.74 (3.92)	6.47 (3.83)	6.13 (3.83)
Inpatient admissions per year of follow-up	0.052	0.054	0.063
Adjusted relative risk	Ref	1.131	1.200
		[1.092, 1.171]	[1.155, 1.246]
[95% CI], p-value^a^		<0.0001	<0.0001
Inpatient days per year of follow-up	0.199	0.217	0.259
Adjusted relative risk	Ref	1.193	1.266
		[1.172, 1.214]	[1.243, 1.290]
[95% CI], p-value^a^		<0.0001	<0.0001

^a^ Models were adjusted for age, sex, BMI, Charlson Index, diabetes, hypertension treatment, hyperlipidemia treatment, osteoarthritis, depression/anxiety treatment, asthma, and GERD.

SD, standard deviation; CI, confidence interval; Ref, reference group; ED, emergency department.

Among the Weight Loss Maintainers, greater magnitudes of weight loss were associated with lower overall healthcare utilization, except for hospitalizations (Table 4). When adjusting for baseline factors, patients with the greatest magnitude of sustained weight loss (>15%) had a lower number of outpatient visits per year. Patients with sustained weight loss >7% had fewer ED visits per year. Patients with sustained weight loss >10% had a higher number of inpatient days per year. There was no association between amount of weight loss and time until ED visit or until hospitalization.

**Table 4:** Poisson Regression for days with healthcare utilization visits per year compared within Weight Loss Maintainers.

Healthcare utilization type by weight loss in WLM group	<7% weight loss	7–10% weight loss	>10–15% weight loss	>15% weight loss
	n = 1,627	n = 2,352	n = 2,947	n = 1,880
Outpatient visits				
Mean total outpatient visits (SD)	37.8 (37.2)	37.6 (36.4)	35.9 (34.9)	35.0 (33.7)
Mean follow-up years (SD)	6.24 (3.89)	6.27 (3.83)	5.99 (3.82)	6.06 (3.79)
Outpatient visits per year of follow-up	6.06	5.99	6.00	5.78
Adjusted relative risk	Ref	0.995	1.002	0.975
		[0.985, 1.005]	[0.992, 1.012]	[0.964, 0.986]
[95% CI], p-value^a^		0.317	0.659	<0.0001
Emergency department (ED) visits				
Mean total ED visits (SD)	0.58 (2.02)	0.49 (2.03)	0.50 (1.63)	0.56 (1.94)
Mean follow-up years (SD)	6.24 (3.89)	6.27 (3.83)	5.99 (3.82)	6.06 (3.79)
ED visits per year of follow-up	0.093	0.078	0.084	0.092
Adjusted relative risk	Ref	0.819	0.813	0.846
		[0.751, 0.893]	[0.749, 0.883]	[0.773, 0.925]
[95% CI], p-value^a^		<0.0001	<0.0001	0.0003
Inpatient (hospital) visits				
Mean total inpatient visits (SD)	0.19 (0.39)	0.18 (0.39)	0.18 (0.39)	0.16 (0.37)
Mean number inpatient days (SD)	1.53 (5.75)	1.57 (6.13)	1.67 (6.59)	1.52 (7.50)
Mean follow-up years (SD)	6.24 (3.89)	6.27 (3.83)	5.99 (3.82)	6.06 (3.79)
Inpatient admissions per year of follow-up	0.031	0.029	0.031	0.026
Adjusted relative risk	Ref	0.979	1.089	0.962
		[0.885, 1.084]	[0.988, 1.200]	[0.861, 1.075]
[95% CI], p-value^a^		0.685	0.085	0.495
Inpatient days per year of follow-up	0.245	0.250	0.279	0.251
Adjusted relative risk	Ref	1.002	1.159	1.083
		[0.952, 1.054]	[1.104, 1.217]	[1.025, 1.143]
[95% CI], p-value^a^		0.954	<0.0001	0.0044

^a^ Models were adjusted for age, sex, BMI, Charlson Index, diabetes, hypertension treatment, hyperlipidemia treatment, osteoarthritis, depression/anxiety treatment, asthma, and GERD.

SD, standard deviation; CI, confidence interval; Ref, reference group; ED, emergency department.

## Discussion

This study demonstrates a robust sample size and long follow-up to assess the real-world clinical impact of weight loss trajectories on disease risk, progression, and prevention, as well as health care utilization. This study found varying associations between obesity and health-related outcomes in a population-based sample. The risk of developing osteoarthritis was lower for patients with sustained weight loss. Cancer-related risk was more nuanced with a positive association of sustained weight loss compared to obesity maintenance over a 10-year period for the rate of cancers in females; the relationship was similar for all cancers and obesity-related cancers. There were no significant correlations between cancer incidence and weight change patterns for males with obesity. There is a growing body of evidence that obesity and cancer are significantly linked. While obesity has been established as risk factor for a subset of cancers [8, 9], there is evidence that obesity may be a risk factor for additional cancer types [35]. Our research is one of the few studies that demonstrates that non-surgical weight loss reduces the risk of developing cancer; the majority of the literature describes associations between reduced cancer incidence and weight loss resulting from bariatric surgery [36–39]. Chlebowski et al. showed reduced cancer incidence in breast cancer for women with non-surgical weight loss [40]. Beyond this, there is little evidence and additional studies, such as ours, are needed to investigate if weight loss can prevent cancer incidence.

Compared to obesity maintenance, weight loss maintenance and weight loss regain were associated with a higher chance of future treatment for depression/anxiety. It has been suggested that there is a complex and bidirectional relationship between obesity and depression, where obesity could increase the risk of depression, and vice versa; this is further complicated in that weight or BMI gain has been associated with anti-depressant use [41, 42].

There was a time-dependent effect on the risk for developing osteoarthritis among Weight Loss Maintainers, with a significant relationship appearing beginning at four years of follow-up with the longest delay seen by the patients with weight loss of >15%. Similar results have been demonstrated by others, where the patients who lost more weight had increased relief of symptoms with larger weight loss outcomes [43, 44]. Meta-analyses of randomized clinical trials of weight loss interventions among patients with osteoarthritis showed that weight loss of 10% or more resulted in moderate-to-large effects on disability, pain, and function [45–47]. There were no statistically significant effects observed for varying magnitudes of weight loss among Weight Loss Maintainers with respect to cancer outcomes, opioid use, or depression outcomes.

Overall healthcare resource utilization (outpatient visits, ED visits, and hospitalizations) was higher for Weight Loss Maintainers and Weight Loss Rebounders compared to Obesity Maintainers though the differences are likely not clinically meaningful. Among Weight Loss Maintainers, those with the greatest weight loss had lower overall healthcare utilization, except for hospitalizations. Healthcare resource utilization did not demonstrate expected trends as it has been shown that obesity is associated with higher healthcare utilization [48, 49]. Future studies will need to explore the relationship between healthcare utilization and obesity. Other studies have shown decreased healthcare costs and utilization associated with weight loss [50, 51]. There are numerous routes for exploration: it is possible that the Weight Loss Maintainers and Weight Loss Rebounders are in better overall health and as such are eligible for elective procedures, such as joint replacement for osteoarthritis; patients who lost weight may have seen their healthcare provider more often to facilitate and maintain weight loss; perhaps these patients are exercising more and have associated injuries with the increased activity. A more detailed analysis that includes pharmacy utilization as well as detailed interventions and activity would be necessary to understand the observed associations with increased healthcare utilization.

While obesity is a major health concern in the U.S. [13], there has been little research into impact of weight loss with regain, and sustained weight loss, particularly at ten years. Current literature highlights the effects of weight loss on specific outcomes or physiological associations, but do not assess weight loss on an epidemiological scale [52–55].

Our retrospective observational study included robust longitudinal data over a median 9.7-year period with over 60,000 patients included in the sample. This study provides an example of large-scale analysis that can inform health outcomes. Prospective data acquisition, including formation of registries for monitoring weight loss efforts could provide deeper insights into the complex nature inherent in evaluating obesity-related outcomes and healthcare utilization patterns. Population level studies are important for understanding prevention and treatment of people with obesity over their lifetime.

Since no information was available on lifestyle behavioral change counseling in the EHR, it is plausible that physicians provided counseling or referred persons with obesity for treatment, consistent with clinical guidelines. Additional research into the techniques that providers use when offering counseling and what, if any, referrals are made, as well as the details of subsequent care is needed. Predictive modeling and observational studies suggest that engaging patients who have personal motivation in obesity management is associated with successful weight loss [56].

### Limitations

There are several limitations to this type of retrospective observational research including misclassification and confounding biases [57]. Data included in the study are limited to visits within the Geisinger Health System, limiting generalizability to other health systems and regions of the U.S. Because the data for this study were based on observations of a primary care cohort receiving standard clinical care, there is some missing or unknown information. For example, a patient without a diabetes diagnosis and no diabetes treatment probably does not have diabetes even if a hemoglobin A1c is not present. When defining outcomes of interest, multiple signals were reviewed to reduce misclassification (e.g., for diabetes we reviewed multiple sources of diagnoses, medication use, and laboratory results). For the majority of the sample, we lack knowledge regarding weight loss strategies used to achieve weight loss in the index period and weight loss may not have been intentional. Many factors could contribute to overall health and the incidence of the outcomes analyzed including exercise, diet, sleep, smoking, alcohol consumption, or engaging in cancer screening or other health services; these factors are not considered in our analysis. The index period is the only time period when weight was monitored. Patients may have experienced weight changes during the observation period that are undocumented that affect the outcomes analyzed. Illness-related weight loss may confound the data related to overall and specific comorbidities in the weight loss group. In older adults, it has been shown that unintentional weight loss is more common than intentional weight loss [58].

The models were adjusted for selected patient characteristics such as baseline weight loss, treatment types, age, sex, and BMI, but there could be a potential for confounding for variables that impact disease trajectory that were not identified and therefore not tested. The generalizability of the study findings may be limited due to the racial/ethnic make-up of the study population. However, our results may be conservative due to the higher prevalence of obesity and cardiometabolic conditions in minority populations.

## Conclusions

In people with obesity, sustained weight loss has greater clinical benefits, such as delayed onset of osteoarthritis and lower cancer incidence, than either regained short-term weight loss or no weight loss at all. Also, higher magnitudes of weight loss delayed onset of osteoarthritis and led to decreased healthcare utilization suggesting we should aim for greater magnitudes of weight loss for people with obesity. Supporting patients in the management of obesity by offering or referring for care and ensuring access to resources and treatments to sustain weight loss long-term will likely further reduce the development of obesity-related conditions in the future.

## Supporting information

S1 Table Cancer types analyzed. (PDF)

S1 Appendix Kaplan-Meier tables. (PDF)
