# Supplementary material for: Effects of sustained weight loss on outcomes associated with obesity comorbidities and healthcare resource utilization
Source: PLoS One. 2021 Nov 3;16(11):e0258545. doi: 10.1371/journal.pone.0258545 (PMC8565747; doi:10.1371/journal.pone.0258545)
Supplement: S1 Table — (PDF) [file pone.0258545.s001.pdf]

S1 Table. Cancer types analyzed

The following table contains counts of the tumor types (categorized by 3-digit ICD-10 code). The results are stratified by sex.

Primary Site	Female	Male	Total
C50 BREAST*	669	11	680
C61 PROSTATE GLAND	0	494	494
C44 SKIN	152	276	428
C34 BRONCHUS AND LUNG	175	248	423
C42 HEMATOPOIETIC/RETICULOENDOTHELIAL SYSTEMS	142	195	337
C54 CORPUS UTERI*	292	0	292
C18 COLON*	125	107	232
C67 BLADDER	36	137	173
C64 KIDNEY*	48	84	132
C25 PANCREAS*	61	65	126
C73 THYROID GLAND*	86	29	115
C77 LYMPH NODES	54	60	114
C80 UNKNOWN PRIMARY SITE	45	35	80
C22 LIVER AND INTRAHEPATIC BILE DUCTS*	26	47	73
C20 RECTUM*	26	38	64
C16 STOMACH*	26	35	61
C15 ESOPHAGUS*	9	51	60
C71 BRAIN	23	34	57
C56 OVARY*	49	0	49
C51 VULVA	46	0	46
C17 SMALL INTESTINE	10	12	22
C49 CONNECTIVE/SUBCUTANEOUS/OTHER SOFT TISSUES	11	11	22
C48 RETROPERITONEUM AND PERITONEUM	17	4	21
C00 LIP (excludes skin of lip C44.0)	5	14	19
C23 GALLBLADDER*	12	7	19
C53 CERVIX UTERI	19	0	19
C09 TONSIL	3	14	17
C01 BASE OF TONGUE	4	12	16
C19 RECTOSIGMOID JUNCTION*	9	6	15
C02 OTHER AND UNSPECIFIED PARTS OF TONGUE	8	6	14
C24 OTHER AND UNSPECIFIED PARTS OF BILIARY TRACT	9	5	14
C32 LARYNX	5	9	14
C07 PAROTID GLAND	6	5	11
C21 ANUS AND ANAL CANAL	9	2	11
C65 RENAL PELVIS	2	9	11
C57 OTHER AND UNSPECIFIED FEMALE GENITAL ORGANS	9	0	9
C41 BONES/JOINTS/ARTICULAR CARTILAGE	5	3	8
C52 VAGINA	8	0	8
C06 OTHER AND UNSPECIFIED PARTS OF MOUTH	2	5	7
C38 HEART, MEDIASTINUM, AND PLEURA	3	4	7
C05 PALATE	2	4	6
C30 NASAL CAVITY AND MIDDLE EAR	3	3	6
C68 OTHER AND UNSPECIFIED URINARY ORGANS	2	4	6

C60 PENIS	0	5	5
C62 TESTIS	0	5	5
C66 URETER	2	2	4
C03 GUM	2	1	3
C10 OROPHARYNX	0	3	3
C31 ACCESSORY SINUSES	1	2	3
C69 EYE AND ADNEXA	1	2	3
C11 NASOPHARYNX	0	2	2
C26 OTHER AND ILL-DEFINED DIGESTIVE ORGANS	1	1	2
C37 THYMUS	2	0	2
C40 BONES, JOINTS AND ARTICULAR CARTILAGE OF LIMBS	1	1	2
C55 UTERUS, NOS	2	0	2
C75 OTHER ENDOCRINE GLANDS	1	1	2
C04 FLOOR OF MOUTH	0	1	1
C08 OTHER AND UNSPECIFIED MAJOR SALIVARY GLANDS	1	0	1
C12 PYRIFORM SINUS	0	1	1
C76 OTHER AND ILL-DEFINED SITES	0	1	1
**Total**	**2,267**	**2,113**	**4,380**

*These cancers were considered obesity-related [1-3]

1. Massetti GM, Dietz WH, Richardson LC. Excessive Weight Gain, Obesity, and Cancer: Opportunities for Clinical Intervention. JAMA. 2017;318(20):1975-6. doi: 10.1001/jama.2017.15519.
2. Centers for Disease Control and Prevention. Cancer and Obesity 2017 [updated October 3, 2017November 17, 2020]. Available from: https://www.cdc.gov/vitalsigns/obesity-cancer/index.html.
3. Steele CB, Thomas CC, Henley SJ, Massetti GM, Galuska DA, Agurs-Collins T, et al. Vital Signs: Trends in Incidence of Cancers Associated with Overweight and Obesity — United States, 2005–2014. MMWR Morb Mortal Wkly Rep. 2017;66:1052-8. doi: DOI: http://dx.doi.org/10.15585/mmwr.mm6639e1External.
