# Supplementary material for: Effects of sustained weight loss on outcomes associated with obesity comorbidities and healthcare resource utilization
Source: PLoS One. 2021 Nov 3;16(11):e0258545. doi: 10.1371/journal.pone.0258545 (PMC8565747; doi:10.1371/journal.pone.0258545)
Supplement: S1 Appendix — (PDF) [file pone.0258545.s002.pdf]

S2 Tables. Kaplan-Meier tables

Kaplan-Meier table for time until any cancer – Females

		Follow-up time (years)									
		**1**	**2**	**3**	**4**	**5**	**6**	**7**	**8**	**9**	**10**
**OM**	%	0.1%	1.0%	1.8%	2.7%	3.6%	4.7%	5.8%	7.0%	8.1%	9.4%
**N=22,136**	N at risk	20,837	18,852	17,030	15,271	13,537	11,801	10,165	8,570	7,058	5,620
**WLR**	%	0.1%	0.7%	1.3%	2.1%	2.9%	3.7%	4.8%	5.6%	6.7%	7.7%
**N=7600**	N at risk	7128	6403	5730	5090	4467	3891	3346	2787	2263	1764
**WLM**	%	0.1%	0.7%	1.6%	2.1%	2.7%	3.3%	4.5%	5.3%	6.1%	6.9%
**N=5,838**	N at risk	5,436	4,819	4,254	3,767	3,283	2,823	2,392	1,977	1,628	1,272

OM, Obesity Maintainers; WLR, Weight Loss Rebounders; WLM, Weight Loss Maintainers.

Kaplan-Meier table for time until any cancer – Males

		Follow-up time (years)									
		**1**	**2**	**3**	**4**	**5**	**6**	**7**	**8**	**9**	**10**
**OM**	%	0.1%	1.2%	2.0%	3.1%	4.2%	5.4%	6.6%	7.9%	9.2%	11.0%
**N=20,398**	N at risk	19,086	17,068	15,274	13,537	11,885	10,276	8,767	7,309	5,990	4,686
**WLR**	%	0.1%	0.8%	1.5%	2.2%	3.0%	4.0%	5.0%	6.2%	7.0%	8.1%
**N=4,627**	N at risk	4,270	3,824	3,392	2,972	2,585	2,206	1,842	1,531	1,240	958
**WLM**	%	0.1%	1.0%	1.9%	3.0%	4.0%	5.0%	6.7%	8.3%	9.6%	10.7%
**N=2,968**	N at risk	2,701	2,313	2,028	1,739	1,464	1,212	1,009	817	651	503

OM, Obesity Maintainers; WLR, Weight Loss Rebounders; WLM, Weight Loss Maintainers.

Kaplan-Meier table for time until obesity-related cancer – Females

		**Follow-up time (years)**									
		**1**	**2**	**3**	**4**	**5**	**6**	**7**	**8**	**9**	**10**
**OM**	%	0.1%	0.7%	1.2%	1.8%	2.4%	3.1%	3.8%	4.6%	5.4%	6.3%
**N=22,136**	N at risk	20,844	18,892	17,086	15,350	13,628	11,902	10,278	8,679	7,173	5,723
**WLR**	%	0.1%	0.4%	0.8%	1.4%	1.9%	2.4%	3.1%	3.6%	4.4%	5.0%
**N=7,600**	N at risk	7,128	6,414	5,751	5,113	4,495	3,917	3,376	2,812	2,288	1,791
**WLM**	%	0.1%	0.4%	1.0%	1.3%	1.7%	2.1%	2.8%	3.3%	3.9%	4.5%
**N=5,838**	N at risk	5,438	4,830	4,272	3,788	3,305	2,843	2,411	1,994	1,640	1,283

OM, Obesity Maintainers; WLR, Weight Loss Rebounders; WLM, Weight Loss Maintainers.

Kaplan-Meier table for time until obesity-related cancer – Males

		**Follow-up time (years)**									
		**1**	**2**	**3**	**4**	**5**	**6**	**7**	**8**	**9**	**10**
**OM**	%	0.0%	0.3%	0.5%	0.8%	1.0%	1.3%	1.6%	1.9%	2.3%	2.9%
**N=20,398**	N at risk	19,093	17,188	15,449	13,762	12,160	10,584	9,098	7,638	6,300	4,980
**WLR**	%	0.0%	0.2%	0.4%	0.6%	0.8%	0.9%	1.0%	1.3%	1.4%	1.9%
**N=4,627**	N at risk	4,272	3,839	3,417	3,000	2,619	2,254	1,895	1,580	1,286	996
**WLM**	%	0.0%	0.5%	0.7%	1.1%	1.2%	1.4%	1.7%	2.0%	2.2%	2.6%
**N=2,968**	N at risk	2,701	2,320	2,043	1,748	1,487	1,233	1,032	837	673	520

OM, Obesity Maintainers; WLR, Weight Loss Rebounders; WLM, Weight Loss Maintainers.

Kaplan-Meier table for time until depression/anxiety

		Follow-up time (years)									
		**1**	**2**	**3**	**4**	**5**	**6**	**7**	**8**	**9**	**10**
**OM**	%	7.8%	13.7%	18.8%	23.8%	28.5%	33.1%	37.4%	41.3%	44.7%	48.4%
**N=25,068**	N at risk	21,871	18,777	16,218	13,877	11,757	9,844	8,128	6,587	5,237	3,965
**WLR**	%	10.3%	17.5%	23.7%	29.9%	35.6%	39.5%	43.0%	46.5%	50.3%	53.4%
**N=5,763**	N at risk	4,879	4,122	3,497	2,907	2,399	1,994	1,619	1,303	1,020	778
**WLM**	%	11.5%	19.2%	25.9%	32.0%	37.1%	42.4%	47.6%	51.8%	54.9%	58.6%
**N=4,031**	N at risk	3,356	2,779	2,313	1,907	1,565	1,250	977	746	594	438

OM, Obesity Maintainers; WLR, Weight Loss Rebounders; WLM, Weight Loss Maintainers.

Kaplan-Meier table for time until osteoarthritis by amount of weight loss among Weight Loss Maintainers

		Follow-up time (years)									
		**1**	**2**	**3**	**4**	**5**	**6**	**7**	**8**	**9**	**10**
**<7%**	%	2.1%	4.1%	6.3%	8.7%	11.7%	13.9%	16.4%	18.3%	20.6%	23.8%
**N=1,177**	N at risk	1,074	916	789	680	576	470	387	312	247	178
**7-10%**	%	1.9%	3.6%	5.8%	8.2%	10.8%	12.5%	15.3%	15.9%	18.2%	21.1%
**N=1,702**	N at risk	1,548	1,341	1,178	1,005	856	713	592	482	392	296
**>10-15%**	%	1.8%	3.8%	5.9%	8.0%	10.0%	11.2%	13.1%	15.1%	16.5%	18.0%
**N=2,171**	N at risk	1,951	1,665	1,445	1,230	1,044	886	709	579	459	348
**>15%**	%	2.0%	4.2%	5.6%	7.6%	8.6%	9.8%	11.5%	12.6%	14.3%	15.4%
**N=1,443**	N at risk	1,308	1,116	956	826	715	605	517	417	335	256
